# High‐Throughput Phenotyping for Revealing Key Morpho‐Physiological Traits for Drought Tolerance in Pea ( *Pisum sativum* and Wild Relatives)

**DOI:** 10.1111/ppl.70863

**Published:** 2026-04-15

**Authors:** Maryam Bagheri, Rick van de Zedde, Diego Rubiales, Carla S. Santos, Marta W. Vasconcelos

**Affiliations:** ^1^ CBQF – Centro de Biotecnologia e Química Fina – Laboratório Associado, Escola Superior de Biotecnologia, Universidade Católica Portuguesa Porto Portugal; ^2^ Wageningen University & Research Wageningen the Netherlands; ^3^ Institute for Sustainable Agriculture, CSIC Córdoba Spain

**Keywords:** architectural traits, drought sensitivity, heritability, pea collection, photosynthetic traits, plant phenotyping, stress sensitivity index

## Abstract

Pea ( *Pisum sativum* ) production is challenged by drought stress. Traditional methods for assessing drought tolerance are limited, and high‐throughput phenotyping (HTP) can facilitate the rapid and automated assessment of plant traits. Herein, 180 *Pisum* spp. accessions were evaluated using an indoor HTP platform under two irrigation treatments, control (70% field capacity) and drought stress (30% field capacity), for 50 days. A combination of digital phenotyping via imaging and manual measurements was used to analyse biomass‐related, architectural, and physiological traits. Drought conditions resulted in significant reductions in biomass‐related traits including fresh weight (47%), total leaf area (43%), and dry weight (41%). In contrast, PSII photochemical efficiency, leaf weight ratio, and solidity showed negative sensitivity index values (ranging from −7% to −1%), indicating comparatively lower sensitivity to drought and suggesting relative stability of these traits under water‐limited conditions. The high heritability value for water use efficiency (0.87) suggests that this parameter may be useful for distinguishing pea's responses to suboptimal soil moisture levels. Principal component analysis (PCA) highlighted patterns of trait variation and associations among biomass‐related traits, such as fresh weight, dry weight, and leaf area, which were sensitive to drought conditions. This suggests that the plants may use a combination of strategies to cope with water limitations. Furthermore, studying the significant variation in drought response among the diverse *Pisum* species and subspecies revealed distinct adaptation strategies. These findings support the development of crops that are resilient to the negative effects of climate change.

## 1 Introduction

In 2024, the global production of peas ( *Pisum sativum* ) was approximately 13–16 million tons of dry peas and 21 million tons of green peas (FAOSTAT 2024). As a widely cultivated legume, peas are a crucial protein source for both human diets and animal feed. Various factors influence production levels, including environmental conditions, market demand, and agricultural practices (Thavarajah et al. 2023).

Drought stress is increasingly impacting pea production, particularly in southern and eastern Europe, where climate change has intensified. Research indicates that extended periods of low rainfall, combined with rising temperatures, have led to significant yield declines in critical agricultural regions, such as Spain, Italy, and Greece (Vicente Serrano et al. 2020; Pinke et al. 2024). For instance, recurring drought in southern Spain has resulted in limited water availability, adversely affecting vegetation and crop yields (Espinosa‐Tasón et al. 2022). Similar trends are evident in Eastern Europe, particularly in Romania and Hungary, where worsening drought conditions further affect crop production (Olesen et al. 2011). Drought stress occurs when water availability is insufficient to meet the plant's physiological needs, resulting in a destructive water deficit (Farooqi et al. 2020). In peas and other legumes, drought stress significantly hinders growth and development, leading to notable yield declines (Abdel‐Hamid and Salem 2021). Drought stress induces a wide range of physiological responses in pea plants that are essential for their survival but are often detrimental to growth and productivity. These responses typically include reduced cell division and proliferation, inhibition of stem elongation and leaf growth, dysregulation of stomatal regulation, and reduced nutrient uptake efficiency (Nadeem et al. 2019). Collectively, these changes reduce water use efficiency (WUE), which represents the plant's ability to convert water into biomass, ultimately leading to reduced crop yield and compromising agricultural performance under water‐limited conditions (Khatun et al. 2021). Therefore, reducing biomass accumulation in legumes such as cowpea (Santos et al. 2020) and common bean (Padilla‐Chacón et al. 2017) is considered as a strategy in response to water limitation.

Furthermore, photosynthesis is a critical process for growth and production and also sensitive to drought stress and reduced water availability, leading to noticeable changes in photosynthetic activity and biomass production in legumes (Juzoń et al. 2019).

To ensure stable pea production under increasingly unpredictable weather patterns, research and adaptation strategies are essential. Wild *P. sativum* representatives, including *P. sativum* subsp. *elatius* and *P. fulvum* , offer greater genetic diversity compared to cultivated species, which can ensure adaptation to a wide range of stresses and improve the objectives of pea breeding programs (Smýkal et al. 2018). As such, breeding programs have gradually focused on developing drought‐tolerant pea varieties while implementing improved irrigation and water management strategies (Bagheri et al. 2023). Marker‐assisted recurrent selection (MARS) and genomic selection have been applied to develop drought‐tolerant pea varieties (Iglesias‐García et al. 2015; Smýkal et al. 2016). However, these methods exceedingly depend on accurate phenotyping, which remains a significant challenge due to its labor‐intensive and time‐consuming nature (Khuimphukhieo and da Silva 2025). To address these issues, high‐throughput phenotyping (HTP) technologies are being investigated as a modern means to accelerate these processes (Cobb et al. 2013; Gondalia et al. 2022).

High‐throughput phenotyping has the potential to accelerate the development of drought‐adapted pea cultivars, offering rapid, accurate, and automated data collection methods (Yang et al. 2020). This approach enables the simultaneous evaluation of numerous genotypes, facilitating early screening of important traits and drastically reducing the time and labor traditionally required for phenotypic evaluations (Yang et al. 2020; Sheikh et al. 2024).

Advanced imaging technologies—including hyperspectral and thermal imaging—provide real‐time tracking of stress indicators, such as leaf temperature and water content, allowing the identification of drought‐tolerant genotypes (Kim et al. 2021). High‐throughput phenotyping platforms have previously demonstrated effectiveness in assessing drought tolerance in various crops, including maize, quinoa (Jaramillo Roman et al. 2021), and wheat, and are now being applied to legumes like peas to evaluate growth dynamics, such as plant height, biomass, chlorophyll content, and convex hull area (Asaari et al. 2019; Correia et al. 2022). Under drought stress, plants typically have smaller canopies and fewer foliage, which significantly reduces the convex hull area, which has been previously reported in faba bean (Belachew et al. 2018), rice (Vishal et al. 2020), and maize (Dar et al. 2021). Vegetation indices obtained from images have previously been used to assess the effects of drought stress in legumes such as common bean (Javornik et al. 2023). Some indices, particularly the Normalized Difference Vegetation Index (NDVI), have been effective in showing drought‐induced reductions in vegetation vigor and chlorophyll content (Trapp et al. 2016). Other pigment‐based indices like the normalized pigment chlorophyll ratio index (NPCI) and the plant senescence reflectance index (PSRI) reflect leaf pigment composition and accelerated senescence under stress conditions (Saglam et al. 2011, Farias et al. 2023). Furthermore, the green leaf index (GLI) has demonstrated a strong correlation with biomass and canopy cover, and is reduced under water‐limited conditions in legumes (Javornik et al. 2023). Despite the advantages of these indices, there remains a need to better understand and identify relationships among traits in the large‐scale drought‐tolerance screening of *Pisum* germplasm.

The large volume of phenotypic data generated through HTP can be integrated with genomic information, including quantitative trait locus (QTL) mapping, to enhance the selection for drought‐tolerant genotypes in breeding programs (Bhat et al. 2020). This combination enables accurate identification of genotype–phenotype correlations, which are critical for efficiently selecting traits associated with drought tolerance in legumes such as pea varieties (Bhat and Yu 2021).

This study aims to utilize digital imaging techniques in conjunction with manually recorded data to evaluate morphophysiological traits in 180 pea accessions under well‐watered (control) and drought‐stress conditions. Automated high‐throughput screening methods were employed to identify sensitive and resistant genotypes and to identify the most relevant traits associated with drought tolerance in pea plants. Specifically, the research will analyse the effects of drought‐stressed (DS) conditions through image‐based and manual trait assessments and the variable responses of accessions in terms of biomass‐related, architectural, and physiological traits as recorded by both digital imaging techniques and manual observations. The overarching ambition is to build a showcase and offer proof/trust for pea breeders to rely more on digital plant phenotyping technologies in their breeding programs and enable them to scale up their capacity to screen many genotypes than current practices.

## 2 Materials and Methods

### 2.1 Plant Material and Growth Conditions

A controlled pot experiment was conducted from March 6 to April 24, 2023, at the Netherlands Plant Eco‐phenotyping Centre (NPEC) greenhouse, Wageningen University and Research in the Netherlands. The study involved 180 *Pisum* spp. accessions [including *Pisum fulvum* (*n* = 6), *P. abyssinicum* (*n* = 2), and various subspecies of *P. sativum* , namely *jomardii* (*n* = 56), *arvense* (*n* = 47), *sativum* (*n* = 37), *humile* (*n* = 10), *elatius* (*n* = 3), and the Indian ecotype of *P. sativum* (*n* = 19)]. Two irrigation treatments were applied: control and drought‐stressed conditions, each replicated three times, resulting in a total of 1080 experimental units.

**FIGURE 1:**
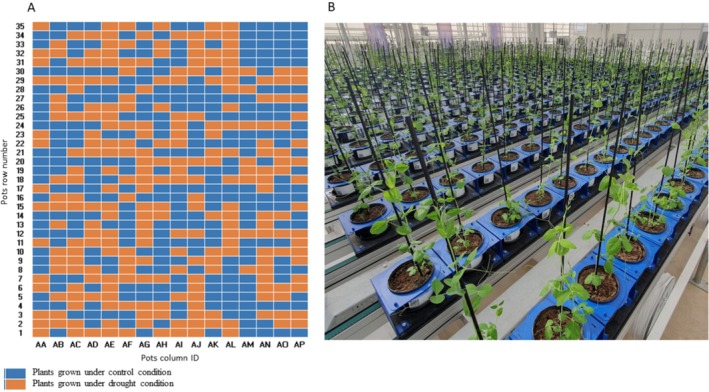
Randomized pot arrangement for control and drought treatments. (A) The pots were placed on conveyor belts, following a completely randomized design, with each rectangular grid representing a pot. Blue ones represent plants grown under control conditions, and orange ones represent those grown under drought conditions. (B) Practical pot arrangement in the greenhouse.

Each plant was grown individually in an approximately 3 L pot (17 cm in diameter and 13 cm in height), filled with potting soil PG MIX 15 + 10 + 20 (Yara Nederland). The pots were placed on conveyor belts within the greenhouse, following a completely randomized design (Figure 1). Greenhouse environmental conditions were strictly controlled, with daytime temperatures maintained at 22°C and night‐time temperatures at 18°C. Relative humidity was set at 60%, and the photoperiod was set to 16 h light.

During the initial growth phase, all pots were watered regularly to maintain 70% field capacity (FC) for both treatment conditions. Drought stress was applied after two weeks, once the pea seedlings had developed four true leaves. At this time, irrigation levels were adjusted to maintain 70% FC for the control group and 30% FC for the drought treatment conditions. Pots were weighed every other day to monitor water needs and ensure precise irrigation levels.

### 2.2 Image‐Derived Plant Traits

HTP was conducted using multiple imaging techniques: RGB side‐view imaging, spectral imaging (SI), and top‐view chlorophyll fluorescence (CF) imaging. The RGB Sideview imaging system, provided by the company SMO BV from Belgium, is equipped with a high‐resolution Allied Vision GT3400C RGB camera, a progressive‐scan‐CCD sensor with bayer filter; 3384 (H) × 2704 (V) pixel with 3.69 μm pixel size. Plants entering the RGB sideview cabinet were stopped on a turn‐table. The rotation of the table was set to make an image at six different angles (0, 60, 120, 180, 240, and 300 degrees). Hence, six images were captured for each plant at each measurement. Each image was masked for plant parts and analysed plant parameters, such as digital biomass, convex hull, height, and solidity. Sideview digital biomass was calculated as the average of six sideview projected areas of plant parts, while height was measured using the pixel height of the plants. The convex hull, which is the smallest area that encloses the entire plant, provides information about the overall shape and size of plant structures. Solidity is the ratio of digital biomass to the convex hull area and represents the compactness of plant structures. The value of solidity ranges from 0 to 1, with higher values indicating a more compact or solid shape. The analysis software for the RGB side‐view imaging has been developed in Python by the NPEC data team, the source is published on Github, accessible via this link: https://github.com/NPEC‐NL/greenhouse_m5.

The topview SI and CF images were captured using the CropReporter camera systems (PhenoVation B.V.). The CropReporterTM consists of a cabinet with a camera system that houses a controller computer, a charge‐coupled device (CCD) camera with an optical filter wheel and a focusing unit with integrated high‐intensity red light‐emitting diodes (LEDs) for excitation of the photosynthesis. All images are captured with the same lens (10 MP 8 mm lens, 200 Lp mm^−1^ resolution, 400–1000 nm spectral range) and CCD camera (1.3 MP, 1296 × 996 pixels), with real 14‐bit signal resolution. Plants were imaged at approximately 140 cm distance from the camera. The output is 16‐bit RAW format. Before the CF measurements, plants were dark‐adapted for 30 min. For the excitation of photosynthesis, 4000 μmolm^−2^ s^−1^ red LED light was used. The integration time for capturing the chlorophyll fluorescence image was 200 μs. The minimum chlorophyll fluorescence (F0) and maximum chlorophyll fluorescence (Fm) images were captured after 10 μs and 800 ms, respectively. Automatic analysis of SI, and CF imaging was performed by Data Analysis (DA)TM software developed by PhenoVation B.V., Wageningen, The Netherlands, which is extensively documented on their website, accessible via https://www.phenovation.com/da‐tutorials.

#### 2.2.1 Imaging and Key Traits

RGB Imaging: RGB images were captured within an imaging cabinet that can rotate pots 360° while making RGB side view pictures from the plant on the turn table equipped with a blue background and LED lighting. The rotation is used to make pictures with different viewing angles. Six side‐view images were taken per plant using a progressive scan CCD sensor (3384 × 2704 pixels). Key traits derived from these images included:

- Side‐View Digital Area (SA): Calculated as the average of six side‐view projected areas of plant parts.
- Convex Hull (CH): The smallest area enclosing the entire plant, providing insights into plant structure and size.
- Digital Biomass (DB): Estimated by averaging the segmented plant profiles from the six images with six different angles.
- Solidity (SOL): Representing plant compactness, calculated as the ratio of DB to CH area.

Chlorophyll fluorescence imaging and spectral imaging measurements were taken using the CropReporter systems (PhenoVation B.V.). These top‐view images were captured with an integrated CCD camera. Key traits are:

- PSII Photochemical Efficiency (PhE) (Fv/fm): Fv/fm was recorded after the plants were dark‐adapted for 30 min and was used as an indicator of the maximum photochemical efficiency of photosystem II and stress‐related photoinhibition, rather than a direct measure of overall photosynthetic performance.
- Water Use Efficiency (WUE) was calculated using the formula:

$$\mathrm{WUE}=\frac{\text{Digital biomass}}{\text{water used}}$$

Several physiological parameters were approximated or calculated using only spectral reflectance in red (RRed), spectral reflectance in green (RGreen), and spectral reflectance in blue (RBlue) derived by spectral imaging (SI):

- Normalized Difference Vegetation Index (NDVI): Used to estimate photosynthetic efficiency, approximated as (Gitelson and Merzlyak 1998)
$$\text{NDVI}\approx \frac{\text{RGreen}-\text{RRed}}{\text{RGreen}+\text{RRed}}$$
- Normalized Pigment Chlorophyll Ratio Index (NPCI): Estimated chlorophyll content using the formula (Merzlyak et al. 1999)
$$\text{NPCI}=\frac{\text{RRed}-\text{RBlue}}{\text{RRed}+\text{RBlue}}$$
- Plant Senescence Reflectance Index (PSRI): Representing leaf senescence and calculated as (Peñuelas et al. 1994)
$$\text{PSRI}=\frac{\text{RRed}-\text{RBlue}}{\text{RGreen}}$$
- Green Leaf Index (GLI): Quantified green parts of the plant using (Louhaichi et al. 2001)
$$\mathrm{GLI}=\frac{2\times \text{RGreen}-\text{RRed}-\text{RBlue}\;}{2\times \text{RGreen}+\text{RRed}+\text{RBlue}\;}$$

### 2.3 Traits Collected Manually

Fifty days after the start of the experiment, the pea plants were harvested at the soil level to measure their fresh weight (FW). Dry weights (DW) were recorded after drying the samples in an oven set to 105°C for 72 h. The total leaf area (TLA) was measured using an LI‐3100C area meter (LI‐COR). Plant height (PH) was recorded in centimetres. Based on these measurements, several key ratios were calculated as follows: Specific Leaf Area (SLA): the ratio of leaf area to leaf dry weight, expressed in cm^2^ g^−1^ DW.

Leaf Weight Ratio (LWR): The ratio of leaf dry weight to the total plant dry weight, expressed in g g^−1^ DW. Leaf Area Ratio (LAR): The ratio of leaf area to total plant dry weight, expressed in cm^2^ g^−1^ DW. The Relative Water Content (RWC) was determined using the formula:

$$R\mathrm{WC}=\frac{\text{Fresh weight}-\mathrm{Dry}\;\text{weight}}{\text{Saturated fresh weight}-\mathrm{Dry}\;\text{weight}}\times 100$$

The sensitivity index (SI) of each trait to drought condition was calculated using this formula.

$$\mathrm{SI}=\frac{\bar{X}c-\bar{X}s}{\bar{X}c}\times 100$$

where $\bar{X}c$ = Mean trait value under drought stress, $\bar{X}s$ = Mean trait value under control condition.

### 2.4 Statistical Analysis

Statistical analyses and data visualizations were conducted using the R programming environment (R Core Team 2022). A two‐way ANOVA was performed for a completely randomized design, followed by Tukey's protected least significant difference (LSD) test to evaluate the statistical significance of differences in trait means among treatments, with a threshold *p*‐value of < 0.05.

The measures of descriptive statistics, box‐plot as well as the Person linear correlation coefficients were used to analyze observed traits. Furthermore, a correlation network map based on the matrix of Person linear coefficients was constructed to visually identify correlation patterns that are not observable in a symmetric correlation matrix (Ursem et al. 2008). In a correlation network map, the traits represent variables as nodes that are connected by edges, whose width is proportional to the strength of the correlation. Based on the REML estimates of the variance components of random terms, the sample‐basis heritability (*h* ^*2*^) of the traits was estimated using the formula equation computed by Holland et al. (2003).

The relationships between traits were further analysed using Pearson correlation coefficients and principal component analysis (PCA) to assess multicollinearity within the dataset.

## 3 Results

### 3.1 Trait Performance Under Drought Stress

Overall, the control conditions consistently yielded the highest means for most of the studied traits (Table 1). However, exceptions were observed: the drought conditions led to decreases in LWR for biomass‐related traits, as well as in PhE and GLI for physiology‐related traits, and in Sol for architectural traits, compared to the control conditions (Table 1). The Sensitivity Index (SI) further revealed the variations in the responses of different traits to drought stress, with both image‐based measures and manual assessments indicating notable drought‐induced effects (Table 1).

**TABLE 1:** Descriptive statistics (mean) and sensitivity indices (SI) to drought for image‐based and manually measured traits of 180 pea accessions grown under control and drought stress conditions.

Traits	Control (mean)	Drought (mean)	SI (%)
Image‐based traits			
DB (Digital biomass)	278.17	188.49	32.24
CH (Convex hull area)	2081.44	1367.50	34.30
Sol (Solidity)	0.15	0.16	−5.77
NPCI (Normalized pigment chlorophyll ratio index)	0.18	0.14	19.8
GLI (Green leaf index)	0.29	0.27	6.93
NDVI (Normalized difference vegetation index)	0.21	0.20	2.86
PSRI (Plant senescence reflectance index)	0.19	0.16	17.28
TA (Top area)	104.58	98.71	5.60
SA (Side area)	173980.09	117979.18	32.18
PhE (PSII photochemical efficiency)	0.77	0.78	−0.94
WUE (Water use efficiency)	2214.52	1691.62	23.61
Manual traits			
FW (Fresh weight)	23.29	12.32	47.10
DW (Dry weight)	4.11	2.41	41.36
PH (Plant height)	123.26	91.44	25.81
TLA (The total leaf area)	818.14	466.32	43.00
SLA (Specific leaf area)	243.53	219.53	9.85
LWR (Leaf weight ratio)	0.30	0.32	−6.66
LAR (Leaf area ratio)	200.30	197.73	1.28
RWC (Relative water content)	75.41	63.348	15.99

Drought stress reduced several traits associated with biomass accumulation (Table 1). Among the manually measured traits, FW decreased by approximately 47%, DW by 41%, TLA by 43%, and PH by 26% (Table 1). Similarly, image‐based traits reflected comparable trends, with DB decreasing by about 32%, CH by 34%, and SA by 32% (Table 1). In contrast, negative sensitivity index (SI) values were observed in three traits: LWR (−6.66%), Sol (−5.77%), and PhE (−0.94%; Table 1).

Analysis of variance disclosed significant effects of both genotype and growth conditions on multiple plant traits (Table S2 and Figure S1). All measured traits showed highly significant genotype effects (*p* < 0.001, except WUE at *p* < 0.01). Different growth conditions (control and drought) also strongly influenced all traits (*p* < 0.001, except TA at *p* < 0.05). Importantly, significant genotype × growth‐condition interactions were detected for most traits (*p* < 0.001), except for GLI and WUE.

Considering the coefficient of variation (CV), most of the studied traits showed relatively high variability with at least 25% in each condition (Table S3). Low (5%–10%) and moderate (10%–20%) variability was found within treatments in RWC, GLI, and NDVI, while very low variability (< 5%) within each condition was observed only in PhE. For most traits, CV was similar across conditions, with slight increases under drought stress. However, fresh FW, DW, and LWR were exceptions, showing lower CV under drought conditions compared to control conditions (Table S3).

**FIGURE 2:**
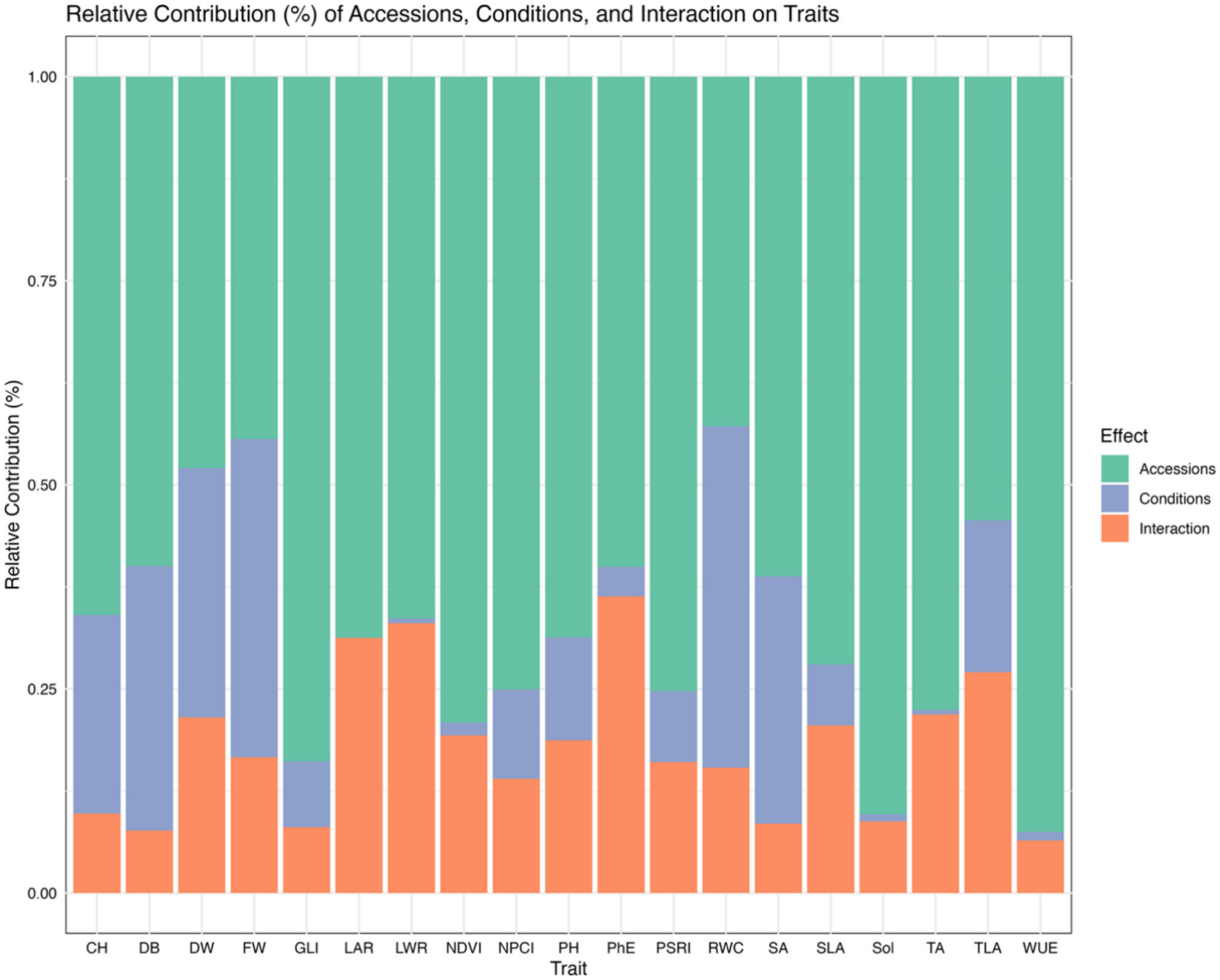
Relative contribution (%) of accessions, conditions, and their interaction on traits. Relative contribution of the variance components to the phenotypic variance of image‐based [Digital Biomass (DB), PSII Photochemical Efficiency (PhE), Top‐area (TA), Side‐View Digital Area (SA), Convex Hull (CH), Solidity (SOL), Water Use Efficiency (WUE), Normalized Difference Vegetation Index (NDVI), Normalized Pigment Chlorophyll Ratio Index (NPCI), Plant Senescence Reflectance Index (PSRI), Green Leaf Index (GLI)] and manually measured [fresh weight (FW), Dry weights (DW), The total leaf area (TLA), Plant height (PH), Specific Leaf Area (SLA), Leaf Weight Ratio (LWR), Leaf Area Ratio (LAR), Relative Water Content (RWC)] traits.

The studied traits showed variable effects of genotype and growth conditions, as well as their interactions (Figure 2 and Table S4). Genotype had the greatest impact on all traits compared to growth condition or their interaction, with the strongest effects observed on water use efficiency (WUE) at 92.5%, solidity (SOL) at 90.4%, green leaf index (GLI) at 83.9%, and leaf area ratio (LAR) at 68.7%. The effect of growth conditions was lesser; however, the highest effect of conditions was observed in some traits related to biomass: relative water content (RWC; 41.8%), fresh weight (FW; 39.0%), digital biomass (DB; 32.4%), dry weight (DW; 30.6%), and side‐view digital area (SA; 30.3%). The interaction effect was dominant in PSII photochemical efficiency (PhE; 36.3%), leaf weight ratio (LWR; 33.1%) as well as in some architectural features such as leaf area ratio (LAR; 31.2%) and total leaf area (TLA; 27%). Heritability (*h* ^*2*^) was generally higher for image‐based traits than for manually measured traits (Table S4). The highest heritability (> 0.90) was for solidity (SOL) and water use efficiency (WUE). The lowest heritability (< 0.50) was obtained for some biomass‐related traits (which were manually collected) such as relative water content (RWC) (0.42), fresh weight (FW; 0.44) and dry weight (DW; 0.47).

### 3.2 Trait Correlations Under Drought and Control Conditions

**FIGURE 3:**
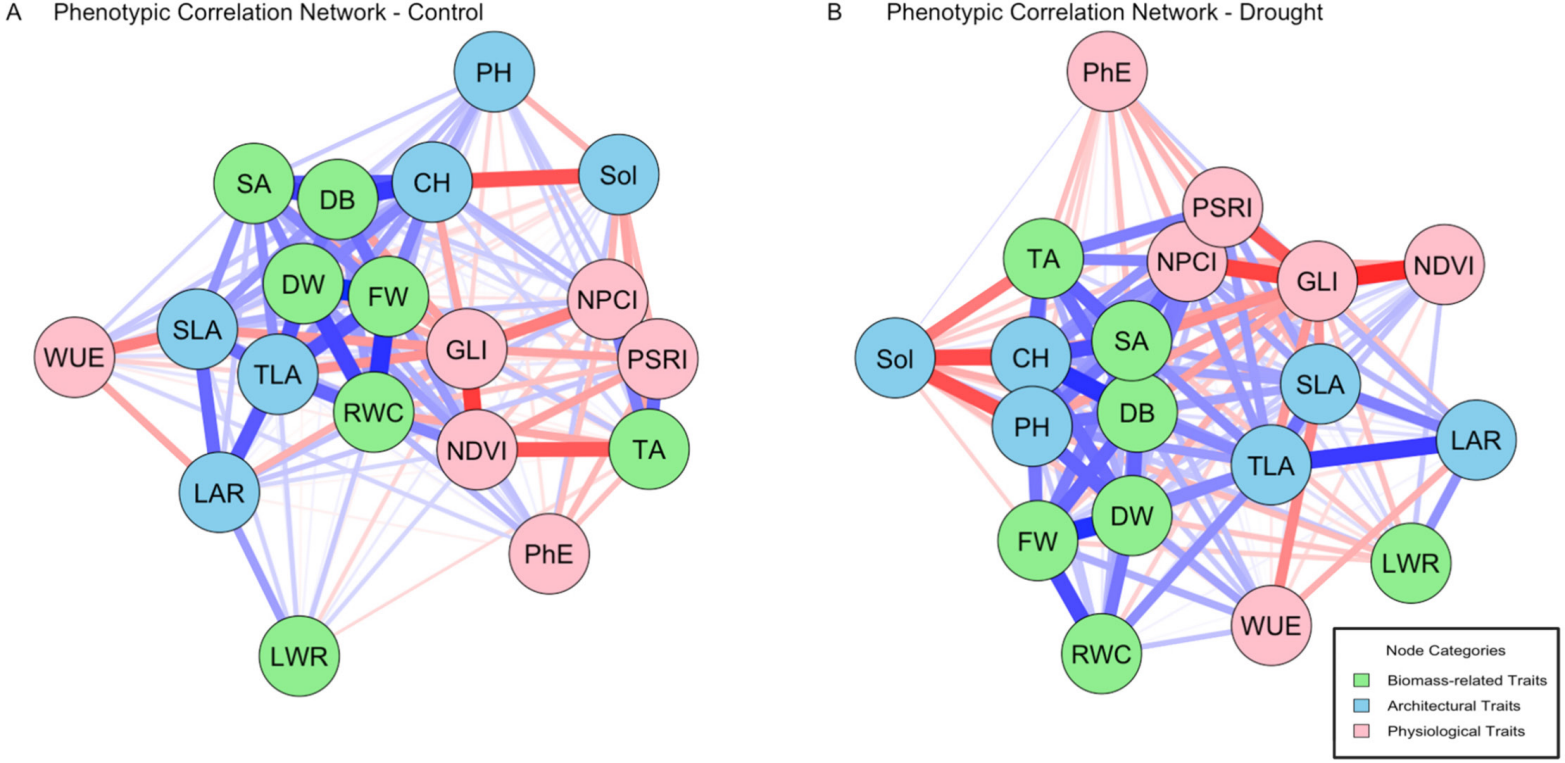
Phenotypic correlation networks of image‐based and manual plant traits under control and drought conditions. Visualization of phenotypic correlation networks showing relationships between image‐based [Digital Biomass (DB), PSII Photochemical Efficiency (PhE), Top‐area (TA), Side‐View Digital Area (SA), Convex Hull (CH), Solidity (SOL), Water Use Efficiency (WUE), Normalized Difference Vegetation Index (NDVI), Normalized Pigment Chlorophyll Ratio Index (NPCI), Plant Senescence Reflectance Index (PSRI), Green Leaf Index (GLI)] and manually measured [fresh weight (FW), Dry weights (DW), The total leaf area (TLA), Plant height (PH), Specific Leaf Area (SLA), Leaf Weight Ratio (LWR), Leaf Area Ratio (LAR), Relative Water Content (RWC)] traits, under control (a) and drought (b) conditions. Green, orange, and purple nodes represent traits related to biomass, architectural features, and physiological traits, respectively. Red connecting lines indicate negative correlations, dark red indicates strong negative correlations, and light red indicates weak negative correlations. Blue connecting lines indicate positive correlations, where dark blue indicates strong positive correlations and light blue indicates weak positive correlations.

Networks visualizing phenotypic (*r*) correlations among all studied traits in the “Control Condition” and “Drought Condition” are given in Figure 3 and Tables S5 and S6.

In control conditions, among the manually measured traits, the highest positive correlations were observed between fresh weight (FW) and two other biomass‐related traits: dry weight (DW) and relative water content (RWC; *r* = 0.91 and 0.81, respectively), as well as dry weight (DW) with relative water content (RWC; *r* = 0.71). Almost the same trend was obtained for drought conditions, where fresh weight (FW) and relative water content (RWC) had a strong positive correlation (*r* = 0.69). However, in drought conditions, plant height (PH) had a significant relationship with convex hull (CH; *r* = 0.73), while it was not observed in the control conditions.

Among the measurements obtained from the image, in both conditions, the highest correlation was shown between the two physiological traits plant senescence reflectance index (PSRI) and normalized pigment chlorophyll ratio index (NPCI; r≤0.98), followed by digital biomass (DB) with convex hull (CH) and side‐view digital area (SA; r≤0.76 and r≤0.68, respectively). In the control condition, a more negative correlation was obtained between Normalized Difference Vegetation Index (NDVI) with green leaf index (GLI) and top area (TA; *r* = −0.74 and −0.63, respectively). Also, the two architectural features solidity (SOL) and convex hull (CH) had a strong negative correlation (*r* = −0.65). Also under drought stress, solidity (SOL) and convex hull (CH) had a significant negative correlation with the same tendency as the control condition (*r* = −0.69). The more negative correlations were observed in dry conditions between some physiological traits green leaf index (GLI) with Normalized Difference Vegetation Index (NDVI), normalized pigment chlorophyll ratio index (NPCI), and plant senescence reflectance index (PSRI; *r* = −0.82, −0.72, and −0.64, respectively).

### 3.3 Principal Component Analysis (PCA)

**FIGURE 4:**
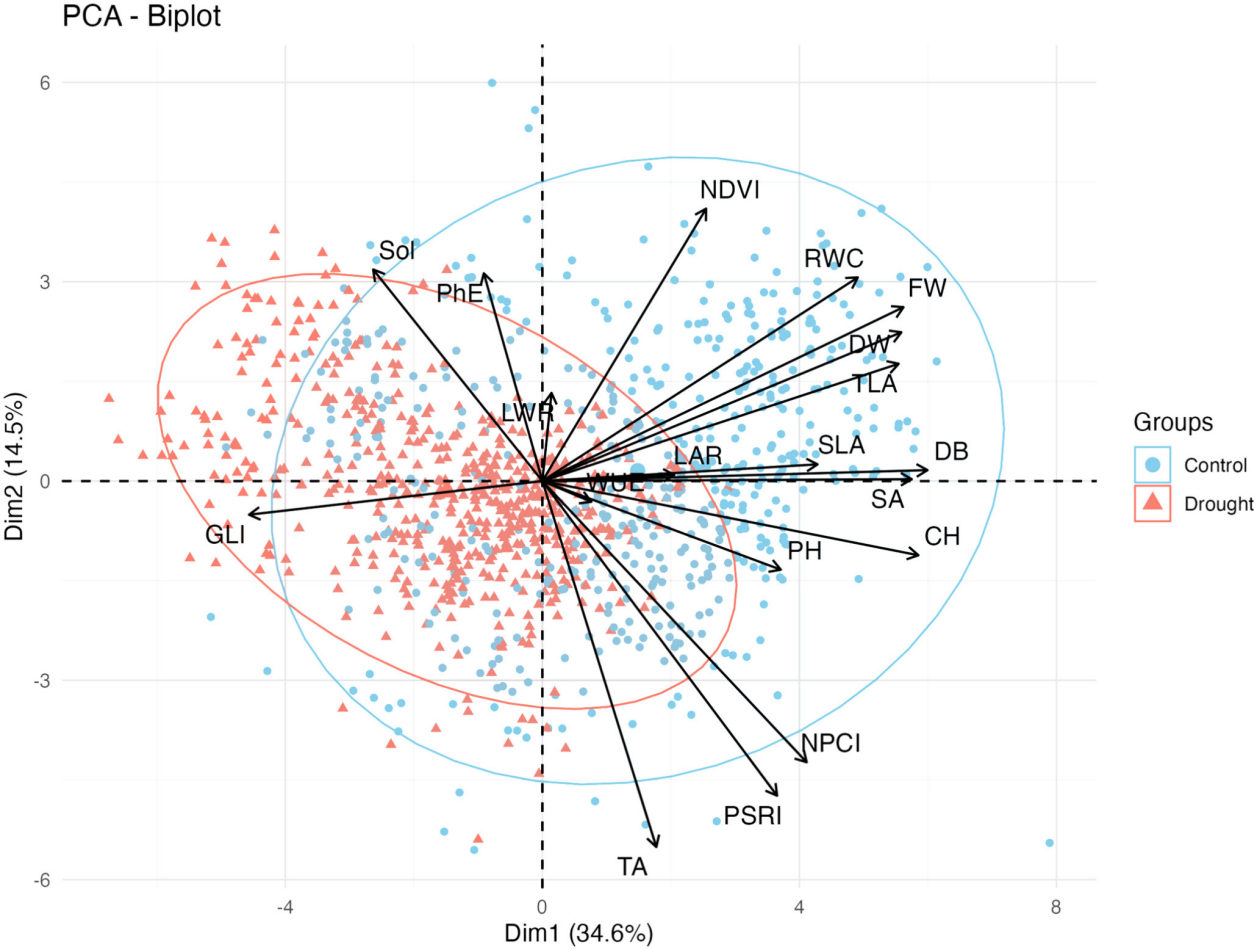
PCA biplot showing separation of plant samples under control and drought conditions. Blue circles (•): Control condition samples and Red triangles (▲): Drought condition samples. Axes: X‐axis: PC1 (Principal Component 1)—explains 34.6% of total variance and Y‐axis: PC2 (Principal Component 2)—explains 14.5% of total variance. Overlapping Areas: Blue shaded area: Distribution range of control samples, Red shaded area: Distribution range of drought samples, and overlap area: Where control and drought conditions share similar characteristics. Arrows: Black arrows showing trait loadings/directions. The length and direction of these arrows indicate how strongly and in what way each trait contributes to the separation between control and drought conditions.

The PCA biplot illustrates patterns of trait variation and associations between control and drought conditions across various traits, with the first two PCA axes explaining approximately 50% of the total variation (Figure 4). The PC1 axis, accounting for almost 35% of the total variance, clearly separates the control and drought conditions, highlighting substantial overall differences. Factors such as PSRI, NDVI, and TA are positioned near to the two architectural traits PH and CH. All biomass‐related traits (except TA), along with certain architectural traits (such as SLA, LAR, and TLA), as well as NDVI, were grouped together. In contrast, traits like PhE and Sol showed distinct separation, indicating significant differences in their behaviour.

### 3.4 The Performance of Different Species of *Pisum* on Selected Agronomic Under Drought Conditions

The experiment included a diverse collection of *Pisum* species, including *Pisum fulvum* , *P. abyssinicum* , and several subspecies of *P. sativum* (named *jomardii*, *arvense*, *sativum*, *humile*, *elatius*, and the Indian ecotype of *P. sativum* ). Given this genetic diversity, it is valuable to evaluate the performance of these species under drought conditions, focusing on their tolerance and response to important agronomic traits.

In this regard, the species‐specific effect on the percentage reduction of dry weight (DW) and plant height (PH), both measured manually under drought conditions, was compared. In addition, water use efficiency (WUE) and PSII Photochemical efficiency (PhE), obtained by image‐based methods, were evaluated. This analysis will help to understand the role of interspecific variation in drought tolerance (or sensitivity) as well as resource utilization under stress conditions in future studies.

**FIGURE 5:**
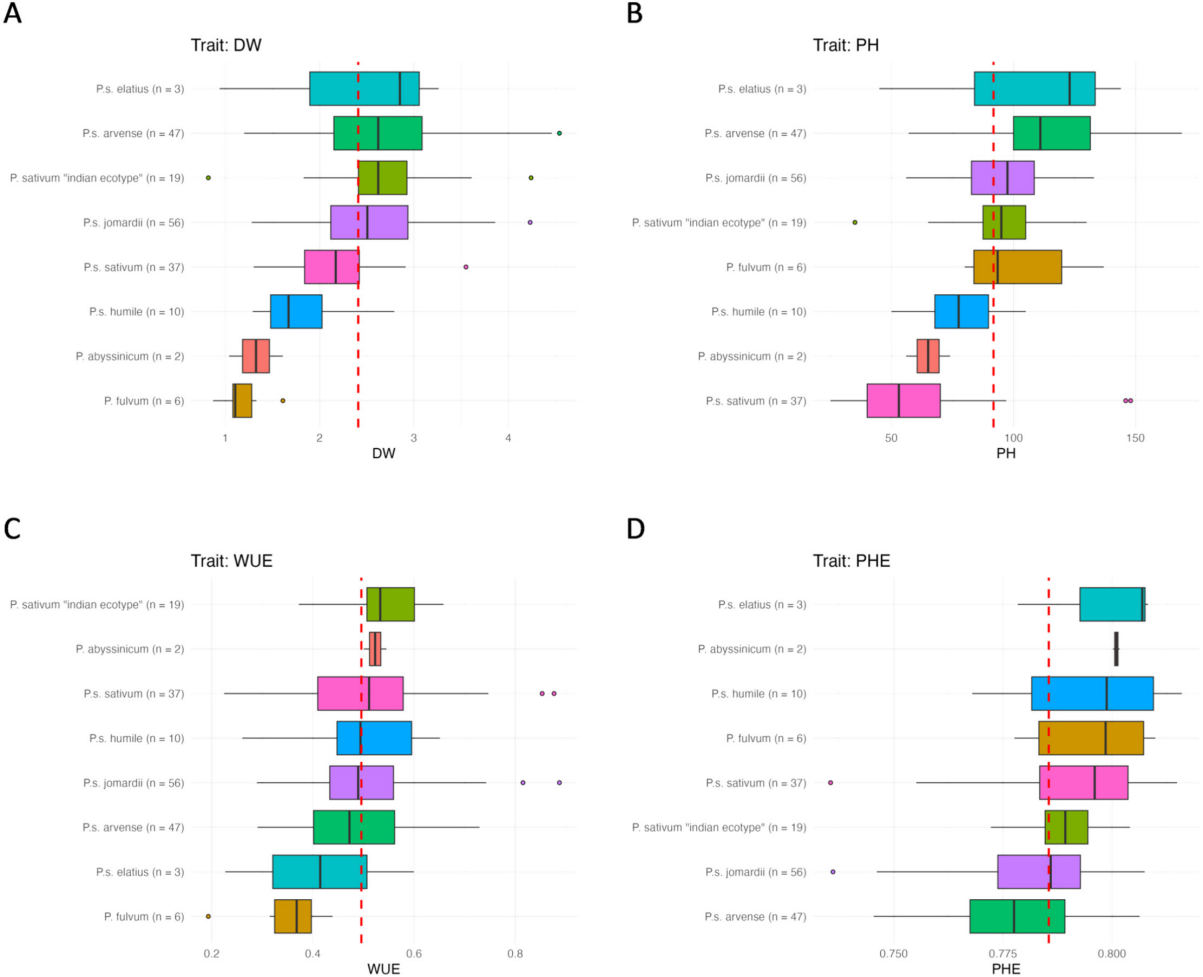
Effect of drought on different plant species. (A) the percentage reduction of dry weight (DW), (B) the percentage reduction of plant height (PH), (C) water use efficiency (WUE) and (D) PSII Photochemical efficiency (PHE), under drought condition, the script calculates the average across all species. The red dashed line shows the overall average of the trait across all species.

*Pisum sativum* subsp. *elatius*, *Pisum fulvum* and *P. sativum* subsp. *humile* demonstrated a smaller decrease in dry weight under drought conditions compared to the control condition (Figure 5a). The greatest reduction in dry weight under drought conditions was observed in *P. sativum* “Indian ecotype”.

A similar trend with dry weight was observed for plant height (Figure 5b), although in this case *P. fulvum* showed the smallest decrease, followed by *P. humile* and then *P. elatius* , while the most significant decrease in plant height under drought condition occurred in *P. sativum* subsp. *Sativum* (Figure 5b).

In terms of water use efficiency (WUE), *P. sativum* “Indian ecotype”, *P. abyssinicum* and *P. sativum* subsp. *sativum* showed the highest performance, respectively, while *P. fulvum* and *P. sativum* subsp. *elatius* showed the lowest water use efficiency under drought conditions (Figure 5c).

In terms of PSII photochemical efficiency, *P. sativum subsp. elatius*, *P. abyssinicum* , *P. sativum subsp. humile*, and *P. fulvum* (in descending order) showed the best performance (Figure 5d). On the other hand, *P. sativum* subsp. *arvense* and *P. sativum* subsp. *jomardii* showed the lowest PSII photochemical efficiency (PhE) under drought conditions, which means a reduced capacity to maintain photosynthetic activity under water limitation conditions.

## 4 Discussion

The expression of traits related to pea growth and stress tolerance was investigated during the vegetative period. These traits were recorded using two different imaging cabinets that detect visible (colour), near‐infrared, fluorescence, and chlorophyll fluorescence. In this study, we focused on traits that are highly informative, showing effects of accessions and conditions, and biologically meaningful, which means they can serve as a proxy for agronomically important traits.

Colour (RBB) imaging can be applied not only to evaluate the growth and other biomass‐related features of plants, but also to assess their health and physiological conditions (Kior et al. 2024). Fluorescence imaging also indicates chlorophyll signalling and can serve as an instrument for detecting stress symptoms (Janka et al. 2018; Elangovan et al. 2023).

In this study, both image‐based features and manually measured attributes were used to assess pea plant traits. It is important to note that all image‐based data were recorded at the final measurement point, aligning with the manual measurements taken at the end of the experiment to ensure consistency. The study aimed to comprehensively evaluate the effects of drought stress on pea plants by examining three key groups of traits: biomass‐related, architectural, and physiological characteristics. Both manual and image‐based measurements provided valuable insights, with each offering complementary data that are equally integral to understanding the plants' overall physiological response to drought.

As expected, drought stress induces significant alterations in plant growth and physiological traits, consistent with prior research in legumes and other crop species (Castillejo et al. 2016; Soureshjani et al. 2019; Godoy Androcioli et al. 2020). In particular, biomass‐related traits (except TA) were more sensitive to applied drought stress among the studied traits.

The negative SI in LWR may be related to reducing transpiration and water loss under drought conditions (Ahmad et al. 2018). Additionally, increasing Sol as an architectural trait suggests potential adaptive responses, such as maintaining plant density under drought stress (Honda et al. 2019). PhE slightly increased under drought, suggesting a relatively stable response under water limitation. Several other traits also showed limited variation (SI < 25%; Table 1), highlighting their relative stability as reliable indicators of drought tolerance for early screening in breeding programs. LAR (1.28%) and NDVI (2.86%) as architectural and physiological traits, respectively, may provide useful information to deal with drought effects.

Analysis of variance component revealed genetic diversity in the studied population and the significant influence of environmental factors on plant phenotypes, highlighting the role of genotype × environment interactions in determining plant traits. Similar observations have been reported by Mansouri et al. (2018) and Xu et al. (2020).

Trait heritability is considered when a new trait is proposed for use in a breeding program to improve crops. Broad‐sense heritability (*h*_*2*_) represents the proportion of total phenotypic variance attributable to genetic factors (Schmidt et al. 2019). In the current study, several architectural traits exhibited slightly higher heritabilities (around 0.60 and greater), while physiological traits (except WUE) and biomass‐related traits showed lower values (≤56and≤36, respectively). Similar reports indicated higher heritability of morphological traits than biomass traits in maize and wheat under drought stress, which may be related to the stronger environmental effects on biomass accumulation (Dodig et al. 2019; Khadka et al. 2020). Environmental conditions accounted for more than 50% of the variation in several biomass‐related traits, including FW, DW, and RWC. In general, heritability estimates of image‐derived data were mostly higher than for manual data, indicating the potential utility of high‐throughput phenotyping for studying plant growth and genetic diversity (Duc et al. 2023).

The relatively high heritability estimate for the WUE (0.87) indicates significant genetic variation among the studied pea accessions and suggests that this trait may help detect variation in response to suboptimal soil moisture levels. We hypothesize that accessions with higher WUE may have lower biomass or more efficient root systems under water‐deficit conditions; however, further investigations are needed to clarify these relationships and evaluate their potential use in pea breeding programs. Similarly, the high heritability of Sol (0.68) in the dry state indicates physiological and structural adjustments. Compact growth forms, characterized by reduced shoot elongation, have been reported as a water‐saving strategy in various crops, including wheat and maize (Khatun et al. 2021; Mohi‐Ud‐Din et al. 2021). These observations are consistent with the concept that several physiological and morphological traits may respond in a coordinated manner under drought conditions (Zia et al. 2021). Similar findings in other legume crops (such as chickpeas and wheat) indicate that multi‐trait interactions become more pronounced under drought stress, reinforcing the importance of studying such integrated responses (Rani et al. 2020; Mohi‐Ud‐Din et al. 2024).

Phenotypic correlations of manually measured traits and automated image‐derived traits can be provided for reliable estimates from a high‐throughput phenotyping approach (Dodig et al. 2021). The moderate positive relationship between DB (obtained from images) and FW and DW (measured manually) observed in the present study was somewhat lower under drought conditions compared with previously reported data in soybean and maize (Dodig et al. 2019; Ranđelović et al. 2023). Early stages of growth usually involve simpler plant structures, which may result in a stronger relationship between estimated digital biomass and actual plant weight. In later stages, leaf overlap, complex branching, or stem elongation reduce the accuracy of biomass measurements and the correlation (Olas et al. 2020).

Increased correlation between traits under drought conditions indicates a coordinated stress response (Ambavaram et al. 2014). However, in the present study, drought conditions were associated with a stronger correlation between the image‐derived CH and manually measured PH. Under drought conditions, plants typically exhibit a more compact and upright growth habit, leading to a stronger correlation between the projected canopy area and manually measured PH (Joshi et al. 2021). Reduced leaf expansion and canopy spread under drought conditions may lead to plant architectures in which height and canopy area vary more closely (Tolley et al. 2020).

The negative correlation obtained between GLI and other pigment indices (NDVI, NPCI, and PSRI), particularly under drought conditions, may be related to differences in their spectral sensitivities and the specific physiological characteristics of the plant they capture (Roman and Ursu 2016). GLI index is derived from the visible spectrum and primarily reflects canopy greenness, while other indices respond strongly to internal leaf structure and carotenoid accumulation (Giovos et al. 2021; Coswosk et al. 2024). Similar results have been reported regarding the sensitivity of GLI under drought stress compared to other pigment indices in common beans (Javornik et al. 2023).

PCA is a statistical method used to reduce the dimensionality of variables and summarize patterns within highly correlated datasets (Gewers et al. 2021). Two‐dimensional PCA biplots help visualize patterns of trait variation and relationships between traits under different environmental conditions. PCA biplot analysis has been widely applied to screen the effects of drought on various legumes (Mohi‐Ud‐Din et al. 2021; Balko et al. 2023; Mohanlal et al. 2023; Basavaraj et al. 2025).

In the present study, the complex distribution of accessions along PC1 and PC2 highlights significant variation within the current population, which can be utilized to identify drought‐tolerant lines of peas for breeding programs. In line with the correlation results, GLI did not cluster with other traits in the PCA analysis, indicating a distinct pattern of variation under the studied conditions. This pattern suggests that GLI may be relatively sensitive to drought stress and could provide useful information for the early detection of drought stress symptoms.

Consistent with the SI results, PhE and Sol clustered together in the PCA biplot, indicating that these traits tend to change together and show slightly higher values under drought conditions. The association indicates that more compact canopies or structurally dense leaves help maintain higher photosynthetic performance, especially under stress conditions (Vico et al. 2023). Additionally, variation in Fv/fm under drought conditions may be associated with differences in PSII photochemical efficiency among accessions (Murchie and Lawson 2013). These results suggest that pea accessions with higher Sol and PhE may adopt a conservative water‐use strategy and maintain their efficiency under stress. The strong clustering of NDVI with biomass‐related traits (except TA) and WUE indicates a close relationship between these features. Similar positive relationship between NDVI and biomass characteristics have also been observed in other legumes such as common beans and soybeans (Barboza et al. 2023; Farias et al. 2023). However, the short WUE arrow in the PCA biplot suggests that WUE is relatively stable across accessions. These observations are particularly useful in high‐throughput phenotyping, where NDVI can serve as a rapid representative for estimating biomass accumulation as well as for discovering drought‐tolerant genotypes.

The clustering of CH, PH, NPCI, PSRI, and TA in the PCA biplot indicates these traits varied together along the same axis of the analysis, reflecting shared variation among plant size and spectral traits. This could mean that growth‐oriented and taller pea accessions are more vulnerable to environmental stresses such as drought compared to smaller plants as were reported in other legumes (Wang et al. 2021; Mansour et al. 2021). This foundational work has laid the groundwork for subsequent experiments and trials in which these findings will be validated at a larger scale (COUSIN project, www.cousinproject.eu).

Also, in this study, significant variation was observed among *Pisum* species and subspecies in response to drought stress, indicating extensive genetic diversity within this genus. Notably, the “Indian ecotype” of *P. sativum* showed the highest dry weight loss under drought conditions (Figure 5a), indicating a high sensitivity of biomass to water limitation. Similarly, *P. sativum* subsp. *sativum* experienced the highest plant height loss (Figure 5b), further supporting the observation that cultivated species have limited adaptive capacity under water stress conditions. These results are consistent with other research, which shows that domesticated genotypes often lose drought‐adapted traits during the breeding process, due to a trade‐off between yield potential and stress tolerance (Annicchiarico et al. 2019). On the other hand, wild relatives such as *P. fulvum* and *P. elatius* maintained more stable growth parameters under drought conditions, possibly due to deeper root systems or conservative growth strategies that evolved under natural selection in arid environments (Smýkal et al. 2018). Another reason may be their inherently smaller stature and slower growth rate, which limit water demand and reduce the apparent impact of drought stress on morphological traits. In other words, the slower, more intensive growth rate, as well as the conservative resource‐use strategy in wild relatives, could provide drought avoidance by reducing transpiration and structural investment. Such performances have been usually reported in some wild legumes adapted to stress conditions (Smýkal et al. 2018; Berger et al. 2017) and could elucidate the relative resilience of these wild species under drought stress.

Regarding physiological traits, water use efficiency (WUE) and PSII photochemical efficiency (PhE) provided additional vision into the response of different species to drought. *P. sativum* ‘Indian ecotype’, *P. abyssinicum* and *P. sativum* subsp. *sativum* showed relatively high water use efficiency (Figure 5c), indicating their ability to maintain biomass accumulation with lower water consumption, an important feature for productivity under drought conditions (Tardieu et al. 2018). In contrast, the species *P. fulvum* and *P. elatius* , although showing low water use efficiency, still exhibited good performance in PSII photochemical efficiency, especially *P. elatius* , *P. abyssinicum* and *P. humile* , indicating that the high‐efficiency photosynthetic apparatus may partially compensate for the low water use characteristics (Figure 5c,d). The ability of these species to maintain photosynthetic activity under drought stress conditions probably helps their tolerance, as photosynthesis is one of the first physiological characters to be affected by water shortage (Qiao et al. 2024). It is noteworthy that *P. arvense* and *P. jomardii* showed the lowest photosynthetic efficiency, which may indicate stomatal closure or photosynthetic pigment degradation under stress (Stefanov et al. 2023).

## 5 Conclusions

This study provides a comprehensive assessment of trait performance across a pea collection under drought conditions, identifying key indicators of drought tolerance. Furthermore, the results of this study show that high‐throughput phenotyping could provide opportunities to discover important drought‐tolerance traits in pea. For greater certainty, the outputs obtained by the images were compared to manual measurements.

The notable reduction observed in biomass accumulation and leaf area traits, in contrast to the moderate flexibility in photosynthetic indices, reveals fundamental trade‐offs between growth factors and survival strategies.

The high SI values obtained in almost all biomass‐related traits indicate their significant sensitivity to drought stress; therefore, these traits can be considered as priority indicators in stress research. On the other hand, the stability of traits such as PSII photochemical efficiency (PhE) and solidity (SOL) under drought conditions indicates their advantages for selecting the most suitable pea lines in breeding programs. Additionally, PCA analysis highlights the crucial role of specific structural and physiological traits in plant adaptation to water stress.

Overall, this study provided comprehensive results for farmers, researchers, and policymakers to identify, predict, and manage the multiple impacts of drought stress on agriculture and natural ecosystems. Continuous monitoring and analysis of these important factors under different climatic conditions will help develop effective strategies for better adaptation to climate and drought challenges.

Additionally, this study reveals significant variation in drought response among diverse *Pisum* species and subspecies, demonstrating their distinct evolutionary histories and adaptive strategies. Some wild relatives, such as *P. elatius* and *P. fulvum* , displayed fairly stable morphological traits under drought stress, likely due to their shorter stature and conservative growth rates. Conversely, some cultivated species, such as *P. sativum* “Indian ecotype” and subspecies *sativum*, showed more reductions in biomass and height under drought conditions, while exhibiting superior water use efficiency, indicating the potential for drought adaptation through efficient water use. Moreover, species with higher PSII photochemical efficiency, including *P. elatius, P. abyssinicum*, and *P. humile* , demonstrated their ability to maintain physiological function under drought stress. These results emphasize the value of integrating morphological and physiological traits for selecting drought‐tolerant pea genotypes and highlight the unrecognized potential of wild germplasm in breeding programs aimed at improving climate resistance in pea and other legumes.

## Author Contributions

M.W.V. and C.S.S. conceived and designed the research. M.B. and R.Z. performed the experiment. M.B. and R.Z. analyzed the data. M.W.V., C.S.S. and D.R. guided the research. M.B. wrote the original draft. M.W.V., C.S.S., D.R. and R.Z. revised the manuscript.

## Funding

This work was supported by Horizon Europe, 101135314; Spanish Ministry of Science and Innovation, MCIN/AEI/10.13039/501100011033; FCT‐Fundação para a Ciência e Tecnologia (Portugal), 2021.08330.BD, 2023.15056.TENURE.039, 2023.15056.TENURE.060.

## Conflicts of Interest

The authors declare no conflicts of interest.

## Supporting information


**Table S1:** Summary of the investigated phenotypic traits in this study.
**Table S2:** Sum of squares measures total variation from the mean. Mean squareis sum of squares divided by degrees of freedom for image‐based traits of 180 pea accessions grown under control and drought stress conditions. F ratio tests factor significance. *p*‐value (Prob > F) indicates result probability.
**Table S3:** Standard deviation (σ) and coefficient of variation (CV) for image‐based and manually measured traits of 180 pea accessions grown under Control and drought stress.
**Table S4:** REML variance components (± standard error of estimates) for random model.
**Table S5:** Matrix for correlation coefficients (r) showing the simple linear relationship among all mesured traits in control condition.
**Table S6:** Matrix for correlation coefficients (r) showing the simple linear relationship among all mesured traits in drought condition.
**Figure S1:** Boxplots summarizing the phenotypic distribution within each condition (control and Drought stress) for all measured traits.

## Data Availability

All data in this study are described in the main text and its Supporting Information. For more data related to this paper, requests should be made to the authors.
