# Supplementary material for: High‐Throughput Phenotyping for Revealing Key Morpho‐Physiological Traits for Drought Tolerance in Pea ( Pisum sativum and Wild Relatives)
Source: Physiol Plant. 2026 Apr 15;178(2):e70863. doi: 10.1111/ppl.70863 (PMC13080420; doi:10.1111/ppl.70863)

High-throughput phenotyping for revealing key morpho-physiological traits in drought tolerance in pea (*Pisum sativum* and wild relatives)

Maryam Bagheri[1], Rick van de Zedde[2], Diego Rubiales[3], Carla S. Santos[1], Marta W. Vasconcelos[1*]

[1] CBQF - Centro de Biotecnologia e Química Fina – Laboratório Associado, Escola Superior de Biotecnologia, Universidade Católica Portuguesa, Rua Diogo Botelho 1327, 4169-005, Porto, Portugal

[2] Wageningen University & Research, Droevendaalsesteeg 4, 6708 PB Wageningen, The Netherlands.

[3] Institute for Sustainable Agriculture, CSIC, Avenida Menéndez Pidal s/n, 14004 Córdoba, Spain

Supplementary Table 1. Summary of the investigated phenotypic traits in this study

	Trait Code	Trait Name	Trait description	Trait category	Camera
Image-based Trait	PhE	Photosynthetic Efficiency	The minimum chlorophyll fluorescence (F0) and maximum chlorophyll fluorescence (Fm) images were captured after 10 µs and 800 ms, respectively,Measured F0 and Fm was used for calculation of the maximum quantum yield for PSII (Fv/Fm).	Physiological	CropReporter
	DB	Digital biomass	Calculated from all side and top view images as sqrt (average plant side area2 x top plant area).	Biomass-related	RGB Sideview
	CH	Convex hull area	Smallest geometrical object without concave parts that covers whole plant from top view, converted in cm2. Provides information on size of the plant.	Architectural	RGB Sideview
	Sol	Solidity	Ratio of projected plant area from top view and the convex hull area from top view. Trait may be a proxy of leaf area index.	Architectural	RGB Sideview
	NDVI	Normalized Difference Vegetation Index	approximated by the peak wavelength for Green and RED reflectance	Physiological	CropReporter
	GLI	Green Leaf Index	calculated by the peak wavelength for Blue, Green and RED reflectance	Physiological	CropReporter
	PSRI	Plant Senescence Reflectance Index	calculated by the peak wavelength for Blue, Green and RED reflectance	Physiological	CropReporter
	NPCI	Normalized Pigment Chlorophyll Ratio Index	calculated by the peak wavelength for Blue and RED reflectance	Physiological	CropReporter
	TA	Top area	Projected plant pixel area from top view, converted in cm2.	Biomass-related	CropReporter
	SA	Side area	Projected plant pixel area from top view, converted in cm2.	Biomass-related	RGB Sideview
	WUE	Water use efficiency	Drigital biomass per plant at the end of the experiment divided by the total amount of water added during experiment.	Physiological	RGB Sideview
Manually measured traits	FW	Fresh Weight	Weight of fresh above-ground biomass , g.	Biomass-related	–
	DW	Dry Weight	Weight of oven dry above-ground biomass,g.	Biomass-related	–
	PH	Plant height	measured by meter,cm.	Physiological	–
	LWR	Leaf Weight Ratio	The ratio of leaf dry weight to the total plant dry weight.	Biomass-related	–
	TLA	The total leaf area	measured using an LI-3100C area meter,cm.	Architectural	–
	LAR	Leaf Area Ratio	The ratio of leaf area to total plant dry weight.	Architectural	–
	RWC	Relative Water Content	determined besaed on FW, DW and aturated Fresh Weight.	Biomass-related	–
	SLA	Specific Leaf Area	The ratio of leaf area to leaf dry weight	Architectural	–

Supplementary Table 2. Sum of Squares measures total variation from the mean. Mean Squareis Sum of Squares divided by Degrees of Freedom for image-based traits of 180 pea Accessions grown under Control and drought stress Conditions.. F Ratio tests factor significance. p-value (Prob > F) indicates result probability.

		Image-based Trait			
		**Sum of Squares**	**Mean Square**	**F**	**p**
DB	Accession	$4.055\times10^{+6}$	$2.265\times10^{+6}$	12.13	<0.001***
	Condition	$2.194\times10^{+6}$	$2.190\times10^{+6}$	1172.08	<0.001***
	Accession ✻ Condition	$5.182\times10^{+5}$	$2.895\times10^{+3}$	1.55	<0.001***
	Residuals	$1.334\times10^{+6}$	$1.868\times10^{+3}$		
Sol	Accession	$1.179\times10^{+3}$	0.012	15.71	<0.001***
	Condition	10.01	0.01	18.61	<0.001***
	Accession ✻ Condition	$1.179\times10^{+3}$	0.001	1135	<0.001***
	Residuals	0.522	7.309×10^{-4}		
CH	Accession	$3.772\times10^{+8}$	$2.107\times10^{+6}$	12.49	<0.001***
	Condition	$1.392\times10^{+8}$	$3.772\times10^{+8}$	825.46	<0.001***
	Accession ✻ Condition	$5.561\times10^{+6}$	$3.107\times10^{+5}$	1.84	<0.001***
	Residuals	$1.203\times10^{+8}$	$1.686\times10^{+5}$		
GLI	Accession	1.18	0.007	12.62	<0.001***
	Condition	0.1	0.108	204.77	<0.001***
	Accession ✻ Condition	0.1	6.091×10^{-4}	1.15	NA
	Residuals	0.37	5.258×10^{-4}		
NDVI	Accession	0.54	0.003	5.35	<0.001***
	Condition	0.01	0.01	18.61	<0.001***
	Accession ✻ Condition	0.13	7.293×10^{-4}	1.31	0.008**
	Residuals	0.4	5.655×10^{-4}		
NPCI	Accession	2.32	0.012	8.08	<0.001***
	Condition	0.33	0.33	210.93	<0.001***
	Accession ✻ Condition	0.43	0.002	1.5	<0.001***
	Residuals	1.14	0.001		
PSRI	Accession	2.62	0.01	7.51	<0.001***
	Condition	0.3	0.3	154.82	<0.001***
	Accession ✻ Condition	0.55	0.003	1.6	<0.001***
	Residuals	1.39	0.001		
TA	Accession	$1.603\times10^{+6}$	8957.101	4.558	<0.001***
	Condition	9603.394	9603.394	4.887	0.027*
	Accession ✻ Condition	868079.883	4849.608	2.468	<0.001***
	Residuals	$1.405\times10^{+6}$	1964.987		
SA	Accession	$1.712\times10^{+12}$	$9.566\times10^{+9}$	11.306	<0.001***
	Condition	$8.473\times10^{+11}$	$8.473\times10^{+11}$	1001.42	<0.001***
	Accession ✻ Condition	$2.381\times10^{+11}$	$1.330\times10^{+9}$	1.572	<0.001***
	Residuals	$6.049\times10^{+11}$	$8.461\times10^{+8}$		
PhE	Accession	0.241	0.001	2.28	<0.001***
	Condition	0.015	0.015	24.63	<0.001***
	Accession ✻ Condition	0.148	8.227×10^{-4}	1.38	0.002**
	Residuals	0.424	5.941×10^{-4}		
WUE	Accession	$8.326\times10^{+7}$	465129.439	1.356	0.004**
	Condition	$7.382\times10^{+7}$	$7.382\times10^{+7}$	215.266	<0.001***
	Accession ✻ Condition	$6.438\times10^{+7}$	359666.162	1.049	NA
	Residuals	$2.469\times10^{+8}$	342941.041		

		Manually measured traits			
		**Sum of Squares**	**Mean Square**	**F**	**p**
FW	Accession	33547	187.4	59.84	<0.001***
	Condition	29490	29489.8	9416.3	<0.001***
	Accession ✻ Condition	12576	70.3	22.43	<0.001***
	Residuals	2236	3.1		
DW	Accession	1115.06	6.23	58.236	<0.001***
	Condition	711.03	711.03	6647.155	<0.001***
	Accession ✻ Condition	500.49	2.8	26.139	<0.001***
	Residuals	76.37	0.11		
PH	Accession	1846185	10314	3.73	<0.001***
	Condition	338116	338116	122.3	<0.001***
	Accession ✻ Condition	502504	502504	2807	NA
	Residuals	973800	2764		
LWR	Accession	9.46	0.05	4.25	<0.001***
	Condition	0.08	0.08	6.7	<0.001***
	Accession ✻ Condition	4.71	0.02	0.02	<0.001***
	Residuals	0.03	0.03		
TLA	Accession	97129895	542625	5.62	<0.001***
	Condition	33281621	33281621	3.45	<0.001***
	Accession ✻ Condition	48307876	48307876	2.79	<0.001***
	Residuals	0	0		
LAR	Accession	6768259	37812	5.26	<0.001***
	Condition	1649	1649	2.29	<0.001***
	Accession ✻ Condition	3075284	17180	2.39	<0.001***
	Residuals	0	0		
RWC	Accession	37857	211	4.68	<0.001***
	Condition	36952	36952	8.17	<0.001***
	Accession ✻ Condition	13585	76	1.67	<0.001***
	Residuals	0.03	0.02		
SLA	Accession	6011756	33585	57.84	<0.001***
	Condition	623815	623815	1074.44	<0.001***
	Accession ✻ Condition	1715181	9582	16.5	<0.001***
	Residuals	414544	581		

Supplementary Table 3. Standard deviation (σ) and coefficient of variation (CV) for image-based and manually measured traits of 180 pea accessions grown under Control and drought stress

	Traits	Condition	σ	CV (%)
Image-based Trait	PhE	Control	0.022	2.8
		Drought	0.016	2
	DB	Control	74.861	26.9
		Drought	54.803	29.1
	CH	Control	731.511	35.1
		Drought	532.311	39
	Sol	Control	0.043	28.8
		Drought	0.049	31.3
	NPCI	Control	0.052	29.2
		Drought	0.049	33.9
	GLI	Control	0.033	11.3
		Drought	0.037	13.7
	PSRI	Control	0.056	28.7
		Drought	0.053	32.8
	NDVI	Control	0.026	12.4
		Drought	0.024	11.5
	TA	Control	50.291	48
		Drought	46.172	46.7
	SA	Control	49614.64	28.5
		Drought	34831.699	29.6
	WUE	Control	0.124	23.7
		Drought	0.127	25.5
	FW	Control	8.926	38.3
		Drought	3.864	31.5
Manually measured traits	DW	Control	1.603	39
		Drought	0.734	30.5
	LWR	Control	0.123	40.8
		Drought	0.106	33.3
	TLA	Control	462.305	56.5
		Drought	240.915	51.7
	LAR	Control	91.875	45.9
		Drought	99.61	50.4
	RWC	Control	7.197	9.6
		Drought	6.715	10.6
	SLA	Control	66.799	27.4
		Drought	63.595	29
	PH	Control	37.152	30
		Drought	30.114	32.8

Supplementary Table 4. REML variance components (± standard error of estimates) for random model of two-way genotype by treatment table and broad-sense heritability (h2)

	Trait	Accessions Variance	Conditions Variance	Total Variance	Heritability (h2)	Accessions	Conditions	Interaction
Image-based Trait	**PhE**	0.000121654	2.75E-05	0.00014919	0.153609146	29.33146198	1.764411844	17.75704293
	**DB**	3462.734988	4.06E+03	7523.418191	0.360583425	50.07942113	27.05398461	6.400110411
	**CH**	322520.9835	259097.7057	581618.6891	0.413514868	54.47583497	20.1031055	8.032617247
	**Sol**	0.001785194	3.54E-05	0.001820551	0.679526239	73.50889007	0.714721613	7.123772885
	**NDVI**	0.000409185	1.72E-05	0.000426397	0.398625965	49.86631686	0.964920981	12.16725648
	**NPCI**	0.001862827	0.000631993	0.002494821	0.436855631	54.77759133	7.986302492	10.20292936
	**PSRI**	0.00207914	0.00056627	0.00264541	0.430040189	53.80326848	6.190927576	11.45485751
	**GLI**	0.001119663	0.000230409	0.001350072	0.565784654	65.71576372	6.319822639	6.305374016
	**TA**	0.59420315	0.009643288	0.603846438	0.382155709	48.43304825	0.360489115	13.65160397
	**SA**	1465600149	1584381464	3049981613	0.367032685	50.36198915	24.94237516	6.978233283
	**WUE**	0.014319429	0.000312573	0.014632002	0.873008038	89.9040491	1.012667053	6.265811653
Manually measured traits	**FW**	28.93447367	54.76920837	83.70368204	0.288237585	43.09242515	37.88086819	16.15435522
	**DW**	0.943395818	1.317944894	2.261340713	0.32417532	46.40371543	29.58986547	20.82804208
	**PH**	1169.503662	647.8864183	1817.39008	0.250570283	39.61255901	7.254769735	10.78195539
	**LWR**	0.007897104	0.00013922	0.008036324	0.592097983	66.36319709	0.582950776	33.05385213
	**TLA**	81814.77167	61786.83152	143601.6032	0.414316671	54.34770905	18.62227727	27.03001368
	**LAR**	5736.641366	0	5736.641366	0.625779635	68.74684584	0.016745221	31.23640894
	**RWC**	33.12686464	68.42552022	101.5523849	0.283580547	42.82699845	41.80395163	15.36904992
	**SLA**	5209.425666	1157.776114	6367.201779	0.595596588	68.58588692	7.116870056	19.56785959

Supplementary Table 5. Matrix for correlation coefficients (r) showing the simple linear relationship among all mesured traits in control condition

	PhE	DB	CH	Sol	NDVI	NPCI	PSRI	GLI	TA	SA	WUE	FW	DW	PH	LWR	TLA	LAR	RWC	SLA
**PhE**	1	0.236346966	0.059393391	0.210362167	0.191833013	-0.248070551	-0.270297835	-0.000265558	-0.238744435	0.068259534	0.162604104	0.213691008	0.196609564	-0.1080518	0.09381579	0.066999789	-0.055165459	0.273390637	-0.021633098
**DB**	0.236346966	1	0.764171907	-0.136885099	0.259981947	0.201350208	0.145601349	-0.366761099	0.039626986	0.744096102	0.214327125	0.547688609	0.535320156	0.210539285	0.050963162	0.446732443	0.166257195	0.381752875	0.393421928
**CH**	0.059393391	0.764171907	1	-0.656270707	0.167959427	0.280615991	0.234544279	-0.340301783	0.177529608	0.648700336	0.205331361	0.475295768	0.470118216	0.31171155	-0.003446912	0.379780725	0.111686897	0.29836695	0.333760363
**Sol**	0.210362167	-0.136885099	-0.656270707	1	0.08959615	-0.291536104	-0.283139861	0.120049207	-0.345740411	-0.226532199	-0.132105491	-0.104936146	-0.141329999	-0.290309471	0.077424781	-0.103758157	-0.025669451	0.017202627	-0.074503263
**NDVI**	0.191833013	0.259981947	0.167959427	0.08959615	1	-0.209743905	-0.359607182	-0.746754331	-0.635813999	0.300298102	-0.07090498	0.385240737	0.368642955	0.015397487	0.159373573	0.35548917	0.157572504	0.456993122	0.267076813
**NPCI**	-0.248070551	0.201350208	0.280615991	-0.291536104	-0.209743905	1	0.981909058	-0.493316226	0.496441693	0.109601538	-0.105424436	-0.099538451	-0.022140995	0.218028062	0.014933816	0.143743406	0.236071394	-0.165639136	0.227550231
**PSRI**	-0.270297835	0.145601349	0.234544279	-0.283139861	-0.359607182	0.981909058	1	-0.348804065	0.572081066	0.060445038	-0.115934741	-0.168103077	-0.081794292	0.20200244	0.004225651	0.086871689	0.213831718	-0.239637378	0.195855289
**GLI**	-0.000265558	-0.366761099	-0.340301783	0.120049207	-0.746754331	-0.493316226	-0.348804065	1	0.227732427	-0.340920867	0.136364877	-0.274826701	-0.313557901	-0.162052138	-0.152500214	-0.414281952	-0.299775448	-0.293215704	-0.393893195
**TA**	-0.238744435	0.039626986	0.177529608	-0.345740411	-0.635813999	0.496441693	0.572081066	0.227732427	1	-0.010752853	0.104335206	-0.166725497	-0.108771691	0.163218535	-0.1425553	-0.094621319	-0.039817089	-0.331775642	-0.049246667
**SA**	0.068259534	0.744096102	0.648700336	-0.226532199	0.300298102	0.109601538	0.060445038	-0.340920867	-0.010752853	1	0.173202608	0.463783761	0.461100071	0.200632709	0.057859258	0.432736132	0.210206662	0.323863311	0.411478477
**WUE**	0.162604104	0.214327125	0.205331361	-0.132105491	-0.07090498	-0.105424436	-0.115934741	0.136364877	0.104335206	0.173202608	1	0.088103748	0.13556461	-0.018403239	-0.050415701	-0.17795638	-0.332130011	0.012427436	-0.488114579
**FW**	0.213691008	0.547688609	0.475295768	-0.104936146	0.385240737	-0.099538451	-0.168103077	-0.274826701	-0.166725497	0.463783761	0.088103748	1	0.91588682	0.230736483	0.050855544	0.659036083	0.049016843	0.814735734	0.299031179
**DW**	0.196609564	0.535320156	0.470118216	-0.141329999	0.368642955	-0.022140995	-0.081794292	-0.313557901	-0.108771691	0.461100071	0.13556461	0.91588682	1	0.259841614	-0.043703442	0.663730756	-0.029948052	0.715861626	0.228089112
**PH**	-0.1080518	0.210539285	0.31171155	-0.290309471	0.015397487	0.218028062	0.20200244	-0.162052138	0.163218535	0.200632709	-0.018403239	0.230736483	0.259841614	1	-0.053868836	0.21631203	0.067394905	0.131890587	0.154392175
**LWR**	0.09381579	0.050963162	-0.003446912	0.077424781	0.159373573	0.014933816	0.004225651	-0.152500214	-0.1425553	0.057859258	-0.050415701	0.050855544	-0.043703442	-0.053868836	1	0.180731663	0.370334249	0.204384078	0.16939366
**TLA**	0.066999789	0.446732443	0.379780725	-0.103758157	0.35548917	0.143743406	0.086871689	-0.414281952	-0.094621319	0.432736132	-0.17795638	0.659036083	0.663730756	0.21631203	0.180731663	1	0.635545835	0.58264733	0.558724364
**LAR**	-0.055165459	0.166257195	0.111686897	-0.025669451	0.157572504	0.236071394	0.213831718	-0.299775448	-0.039817089	0.210206662	-0.332130011	0.049016843	-0.029948052	0.067394905	0.370334249	0.635545835	1	0.123179595	0.576296008
**RWC**	0.273390637	0.381752875	0.29836695	0.017202627	0.456993122	-0.165639136	-0.239637378	-0.293215704	-0.331775642	0.323863311	0.012427436	0.814735734	0.715861626	0.131890587	0.204384078	0.58264733	0.123179595	1	0.259301676
**SLA**	-0.021633098	0.393421928	0.333760363	-0.074503263	0.267076813	0.227550231	0.195855289	-0.393893195	-0.049246667	0.411478477	-0.488114579	0.299031179	0.228089112	0.154392175	0.16939366	0.558724364	0.576296008	0.259301676	1

Supplementary Table 6. Matrix for correlation coefficients (r) showing the simple linear relationship among all mesured traits in drought condition

	PhE	DB	CH	Sol	NDVI	NPCI	PSRI	GLI	TA	SA	WUE	FW	DW	PH	LWR	TLA	LAR	RWC	SLA
**PhE**	1	-0.212769668	-0.193209103	0.130138721	-0.024067887	-0.283724055	-0.286892669	0.182229911	-0.250821858	-0.242851054	0.060449693	-0.065927042	-0.059643787	-0.179639757	0.045099341	-0.090190005	-0.080383795	0.081127314	-0.277939193
**DB**	-0.212769668	1	0.825486697	-0.30781432	0.105464163	0.508718271	0.494530381	-0.368539399	0.562610572	0.78867615	0.300299545	0.588305377	0.601540316	0.553464641	-0.037772891	0.457117917	0.132887467	0.32152187	0.348998485
**CH**	-0.193209103	0.825486697	1	-0.694245056	0.114110963	0.442353302	0.419273	-0.336545083	0.595103985	0.685681692	0.214292937	0.529926829	0.566656822	0.738409379	-0.114175038	0.39992178	0.076281281	0.226050877	0.363268192
**Sol**	0.130138721	-0.30781432	-0.694245056	1	0.043330333	-0.201519328	-0.18716068	0.087009126	-0.516141087	-0.35039836	-0.096944565	-0.175282058	-0.245493479	-0.669962782	0.213997784	-0.15353021	-0.00084076	0.047187079	-0.193591903
**NDVI**	-0.024067887	0.105464163	0.114110963	0.043330333	1	0.207620177	0.102499913	-0.825619207	-0.31083864	0.141940742	-0.135791976	0.137856014	0.1995227	0.178058211	0.214294728	0.237865211	0.120065726	0.194620319	0.271679056
**NPCI**	-0.283724055	0.508718271	0.442353302	-0.201519328	0.207620177	1	0.986969216	-0.723061554	0.4712407	0.575412106	0.033034412	0.18545198	0.273797843	0.406646564	0.151881535	0.399580036	0.27569427	0.06867125	0.357508944
**PSRI**	-0.286892669	0.494530381	0.419273	-0.18716068	0.102499913	0.986969216	1	-0.641948892	0.497255037	0.552597516	0.012204239	0.148494054	0.239826382	0.375411212	0.157026232	0.388884448	0.291784101	0.03727551	0.351073876
**GLI**	0.182229911	-0.368539399	-0.336545083	0.087009126	-0.825619207	-0.723061554	-0.641948892	1	-0.052238865	-0.43278676	0.077759301	-0.204969899	-0.299051787	-0.361533975	-0.238836195	-0.399028635	-0.244344543	-0.17711606	-0.39976758
**TA**	-0.250821858	0.562610572	0.595103985	-0.516141087	-0.31083864	0.4712407	0.497255037	-0.052238865	1	0.603428866	0.253664676	0.279574583	0.312261795	0.507842713	-0.224592766	0.191194478	0.001062513	0.013333643	0.174756564
**SA**	-0.242851054	0.78867615	0.685681692	-0.35039836	0.141940742	0.575412106	0.552597516	-0.43278676	0.603428866	1	0.248241138	0.503835881	0.508072957	0.537843544	-0.017460326	0.432177214	0.152282867	0.271042707	0.360581092
**WUE**	0.060449693	0.300299545	0.214292937	-0.096944565	-0.135791976	0.033034412	0.012204239	0.077759301	0.253664676	0.248241138	1	0.315148127	0.353490607	0.152488914	-0.118058917	-0.086594448	-0.306693359	0.235234857	-0.460149236
**FW**	-0.065927042	0.588305377	0.529926829	-0.175282058	0.137856014	0.18545198	0.148494054	-0.204969899	0.279574583	0.503835881	0.315148127	1	0.855896654	0.524321618	-0.191102493	0.455978067	-0.072219999	0.696705907	0.204057722
**DW**	-0.059643787	0.601540316	0.566656822	-0.245493479	0.1995227	0.273797843	0.239826382	-0.299051787	0.312261795	0.508072957	0.353490607	0.855896654	1	0.594409642	-0.226077773	0.456038546	-0.105487058	0.530041112	0.151837909
**PH**	-0.179639757	0.553464641	0.738409379	-0.669962782	0.178058211	0.406646564	0.375411212	-0.361533975	0.507842713	0.537843544	0.152488914	0.524321618	0.594409642	1	-0.259796917	0.392126433	0.03607864	0.2150656	0.350534459
**LWR**	0.045099341	-0.037772891	-0.114175038	0.213997784	0.214294728	0.151881535	0.157026232	-0.238836195	-0.224592766	-0.017460326	-0.118058917	-0.191102493	-0.226077773	-0.259796917	1	0.122524262	0.414278212	0.028836787	0.064259553
**TLA**	-0.090190005	0.457117917	0.39992178	-0.15353021	0.237865211	0.399580036	0.388884448	-0.399028635	0.191194478	0.432177214	-0.086594448	0.455978067	0.456038546	0.392126433	0.122524262	1	0.757294368	0.430689403	0.550762354
**LAR**	-0.080383795	0.132887467	0.076281281	-0.00084076	0.120065726	0.27569427	0.291784101	-0.244344543	0.001062513	0.152282867	-0.306693359	-0.072219999	-0.105487058	0.03607864	0.414278212	0.757294368	1	0.046864883	0.489769849
**RWC**	0.081127314	0.32152187	0.226050877	0.047187079	0.194620319	0.06867125	0.03727551	-0.17711606	0.013333643	0.271042707	0.235234857	0.696705907	0.530041112	0.2150656	0.028836787	0.430689403	0.046864883	1	0.107887916
**SLA**	-0.277939193	0.348998485	0.363268192	-0.193591903	0.271679056	0.357508944	0.351073876	-0.39976758	0.174756564	0.360581092	-0.460149236	0.204057722	0.151837909	0.350534459	0.064259553	0.550762354	0.489769849	0.107887916	1

Supplementary Figure 1. Boxplots summarizing the phenotypic distribution within each condition (control and Drought stress) for all measured traits.

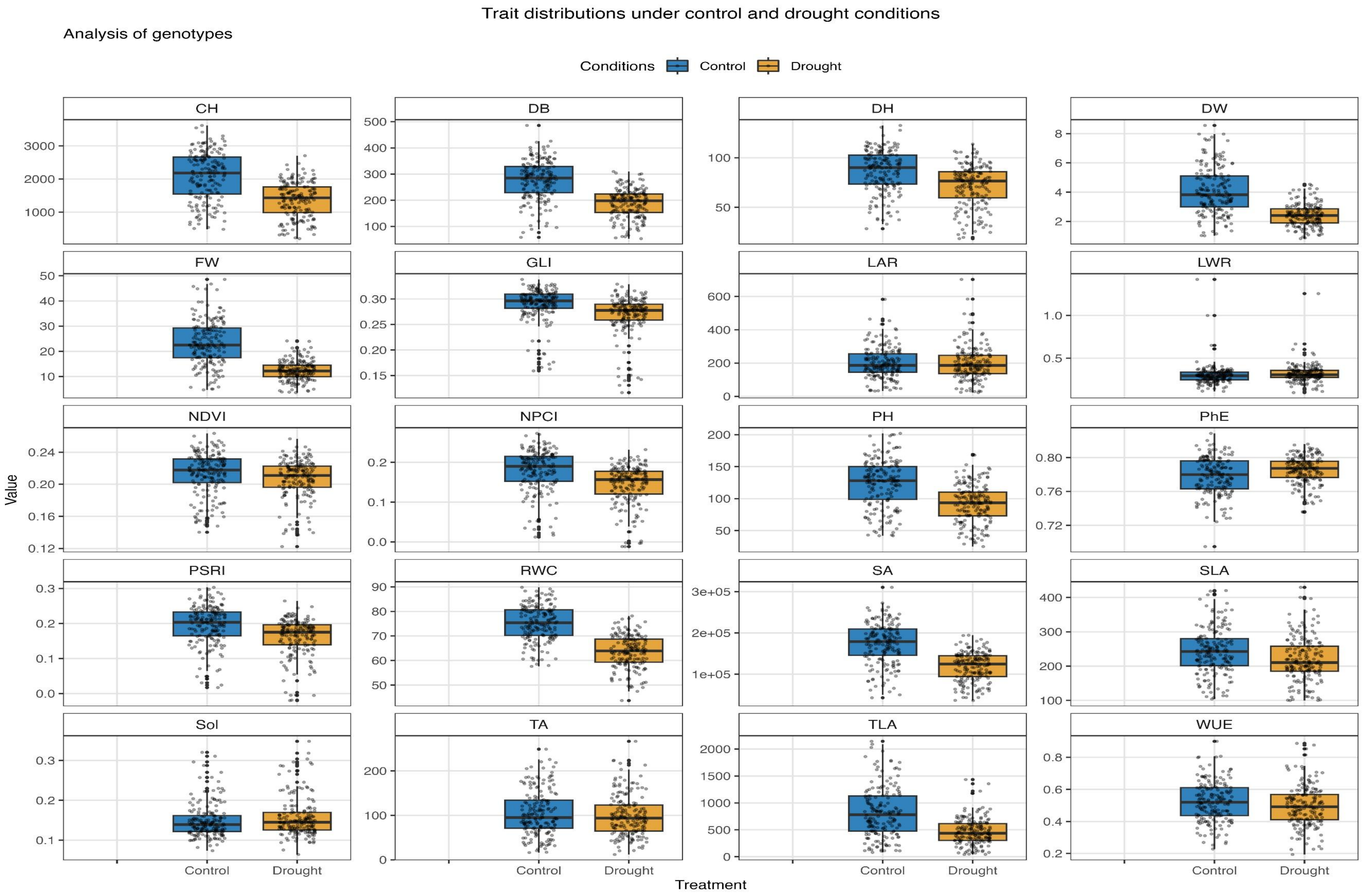

Supplement: Supplementary file 1 — Table S1: Summary of the investigated phenotypic traits in this study. Table S2: Sum of squares measures total variation from the mean. Mean squareis sum of squares divided by degrees of freedom for image‐based traits of 180 pea accessions grown under control and drought stress conditions. F ratio tests factor significance. p‐value (Prob > F) indicates result probability. Table S3: Standard deviation (σ) and coefficient of variation (CV) for image‐based and manually measured traits of 180 pea accessions grown under Control and drought stress. Table S4: REML variance components (± standard error of estimates) for random model. Table S5: Matrix for correlation coefficients (r) showing the simple linear relationship among all mesured traits in control condition. Table S6: Matrix for correlation coefficients (r) showing the simple linear relationship among all mesured traits in drought condition. Figure S1: Boxplots summarizing the phenotypic distribution within each condition (control and Drought stress) for all measured traits. [file PPL-178-e70863-s001.pdf]
